# Value of HE4 Combined with Cancer Antigen 125 in the Diagnosis of Endometrial Cancer

**DOI:** 10.12669/pjms.334.12755

**Published:** 2017

**Authors:** Chunhua Dong, Ping Liu, Chao Li

**Affiliations:** 1 Chunhua Dong, Department of Gynaecology, Binzhou People’s Hospital, Shandong, 256610, China; 2 Ping Liu, Office of Binzhou People’s Hospital, Library of Binzhou People’s Hospital, Binzhou People’s Hospital, Shandong, 256610, China; 3 Chao Li, Department of Gastroenterology, Binzhou People’s Hospital, Shandong, 256610, China

**Keywords:** Endometrial cancer, Human Epididymis Protein 4, Cancer antigen 125

## Abstract

**Objective:**

To investigate the clinical significance of human epididymal secretory protein E4 (HE4) in combination with cancer antigen 125 (CA125) in the diagnosis of endometrial cancer.

**Methods:**

One hundred and fifty patients with endometrial cancer who were admitted to Binzhou People’s Hospital, Shandong, China, between June 2013 and July 2014, were enrolled and set as an endometrial cancer group; another one hundred patients with benign uterine diseases and one hundred healthy females were also enrolled. The serum was collected from the subjects for the detection of HE4 level. The level of CA125 was detected using electrochemiluminescence assay (ELISA). Receiver Operating Characteristic (ROC) curve was drawn to analyze the cutoff points of HE4 and CA125 levels for the diagnosis of endometrial cancer. The diagnostic efficacy based on the detection of the two indexes separately and jointly was evaluated.

**Results:**

The area under curve (AUC) for diagnosis of endometrial cancer based on HE4 was superior to that based on CA125 (0.819 vs 0.757). The optimal diagnosis cutoff point of HE4 and CA125 on the ROC curves was 92.21 pmol/L and 31.32KU/L, respectively. The sensitivity, Youden index, coincidence rate and negative predicted value of diagnosing endometrial cancer with HE4 in combination with CA125 (73.2%, 0.641, 83.5% and 83.4%) were significantly higher than those of diagnosing endometrial cancer with the two indexes separately. The ROC-AUC value of serum HE4 and CA125 was 0.749 and 0.528 respectively, much lower than that of HE4 in combination with CA125 (0.794; P<0.05).

**Conclusion:**

Serum HE4 and CA125 are the ideal marker combination for the diagnosis of endometrial cancer. HE4 combined with CA125 is beneficial to the diagnosis of endometrial cancer; hence it is worth promotion in clinical practice.

## INTRODUCTION

Endometrial cancer, one of the most commonly seen malignant tumors in female reproductive tract, histologically originates from endometrial glands.^1^ The five-year survival rate of endometrial carcinoma in situ is 95%, but decreased to 44% after distant metastasis occurs. Endometrial cancer is frequently seen in middle-aged and elderly females. But with the improvement of economic condition and the transformation of life style, more and more young females tends to develop the disease, which severely threatens the reproductive health of females.^2^,^3^ About 70% of endometrial cancer could be diagnosed in early stage; the prognosis was favorable if it was diagnosed in early stage and poor if diagnosed in late stage.^4^ Therefore, early discovery, diagnosis and treatment is an important approach for improving the survival rate of patients with endometrial cancer.

Currently, diagnostic curettage and imaging are the major methods for diagnosing endometrial cancer; however, they had indistinctive advantages and cannot screen population with risks through general investigation.^5^ The promotion of tumor markers in the early diagnosis of clinical malignant tumors makes the early diagnosis of endometrial cancer possible.^6^ Serum cancer antigen 125 (CA125), a kind of high-molecular-weight protein expressed in coelomic epithelium in embryonic development, has been extensively applied for the diagnosis and monitoring of malignant tumors in organs such as ovary, mammary gland, digestive tract and respiratory tract; it is the most commonly used tumor marker for the diagnosis of ovarian cancer currently. However, it has limited clinical application value as its expression in early stage of tumor is insufficient in early stage of tumor. CA125 only shows an increase in less than half patients with early-stage tumor and varying degrees of increase in many benign diseases.^7^,^8^ HE4 distributed on chromosome 20q and with a length of 12 kb was discovered in acidoglycoprotein in human epididymal epithelium. It is a kind of protease inhibitor which is associated with innate immunity of body and sperm maturation. HE4 is mainly distributed in germinal epithelium and expressed in oviduct epithelium, endometrial glands and Bartholin’s gland of females, but its content in the serum of normal people is quite low.^9^,^10^

In recent years, the application of HE4 in combination with CA125 in the diagnosis of endometrial cancer has been questioned by experts. Our objective was to study the feasibility and accuracy of HE4 and CA125 as the markers of the diagnosis of endometrial cancer of patients especially high risk patients, this study retrospectively analyzed the levels of HE4 and CA125 of 150 patients with endometrial cancer.

## METHODS

One hundred and fifty patients who were pathologically confirmed as primary endometrial cancer in the Binzhou People’s Hospital from June 2013 to June 2014 were selected and set as endometrial cancer group; 50% of them suffered from menopause. They aged from 38 to 75 years old (average 53.2±15.3 years old). According to the criteria of Federation Internationale of Gynecologie and Obstetrigue (FIGO) (2009), there were 73 cases of stage I, 34 cases of stage II, 30 cases of stage III, and 13 cases of stage IV. In addition, one hundred patients with endometrial hyperplasia were set as uterine benign diseases group; they aged from 31 to 69 years old (average 52.8±16.6 years old). One hundred healthy females were set as control group; they aged from 30 to 67 years old (average 51.8±16.8 years old). The age composition of the three groups had no statistically significant difference (P>0.05). The enrolled patients signed informed consent, and this study has been approved by the ethics committee.

### Inclusion and exclusion criteria

Patients who were confirmed by histopathology, did not undergo uterus surgery three months before admission, and did not receive any systemic treatment were included. Those who had primary or malignant tumors in other organs or had severe liver and kidney function deficiency or females at lactation period were excluded.

## METHODS

Blood specimens were collected from the patients in the endometrial cancer group and the benign uterine diseases group prior to treatment. 5 ml of fasting blood was extracted from each subject in the morning. Serum was separated from the blood specimens for the detection of HE4 and CA125.

The level of serum CA125 was detected with a USA NAXSY biochemical analyzer and the matched reagents using electrochemiluminescence assay. The level of serum HE4 was detected with a kit (Wuhan Boster Biological Engineering Co., Ltd., Hubei, China) and CODA microplate reader (BIO-RAD, US) using enzyme-linked immune-sorbent assay (ELISA).

Pathological diagnosis was regarded as the gold standard. The malignant group was determined as true positive. Receiver Operating Characteristic (ROC) curves were drawn to analyze the cutoff points of HE4 and CA125 levels for the diagnosis of endometrial cancer. Moreover, the diagnostic efficacy based on the detection of two indexes separately and jointly was evaluated.

The reference scope of the serum HE4 and CA125 was less than 86 pmol/L and less than 35 U/mL, respectively. The level higher than the critical value was defined as positive. Duplicable test was performed in the detection of HE4 in combination with CA125. The result of the combination detection was determined as positive if the level of any of them was positive.

Data was analyzed using SPSS 20.0. Enumeration data (percentage) were compared between groups using Chi-square test. Difference was considered as statistically significant if P<0.05.

## RESULTS

### Analysis of ROC curves of diagnosis of endometrial cancer based on HE4 and CA125

**Fig. 1:**
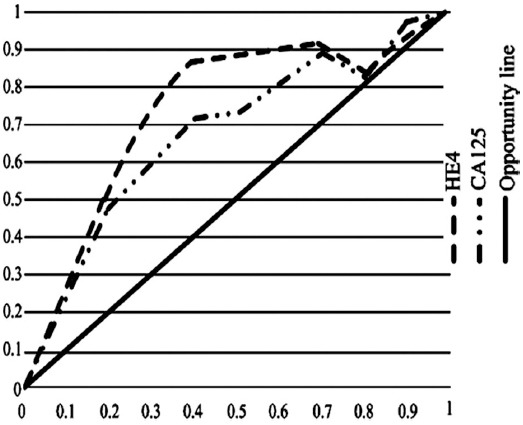
ROC curves for diagnosis of endometrial cancer based on HE4 and CA125.

The subjects in the benign uterine diseases group and healthy control group were determined as negative, while the patients in the malignant group were determined as positive. ROC curves were drawn for the diagnostic sensitivity and specificity of endometrial cancer based on different cutoff points of HE4 and CA125 (Fig. 1).

### Comparison of the parameters of curves for diagnosis based on HE4 and CA125

AUC of diagnosis based on HE4 was superior to that based on CA125 (0.819 vs 0.757). The cutoff points corresponding to the largest Youden index of the ROC curves, i.e., 0.518 for HE4 and 0.455 for CA125, were determined as optimal (Table-I).

**Table-I:** Comparison of parameters of HE4 and CA125 ROC curves

*Item*	*AUC*	*Standard deviation*	*P*	*95% confidence interval*	*Optimal cutoff points*
CA125	0.819	0.26	<0.05	0.724~0.933	92.21 pmol/L
HE4	0.757	0.25	<0.05	0.709~0.901	31.32 KU/L

AUC: Area under curve.

### Comparison of negative and positive conditions of HE4 and CA125 under the optimal cutoff points

When the optimal cutoff points confirmed before were applied, there were differences in the number of negative and positive cases of HE4 and CA125 between the three groups (P<0.05). In the comparison of the endometrial cancer group with the benign uterine diseases group and the healthy control group, the difference was statistically significant (P<0.05). The difference between the benign uterine diseases group and the healthy control group suggested no statistical significance (P>0.05) (Table-II).

**Table-II:** Comparison of the negative and positive conditions of CA125 and HE4 under the optimal cutoff points.

*Group*	*N*	*CA125*		*HE4*	

		*Negative*	*Positive*	*Negative*	*Positive*
Endometrial cancer group	150	73	77	65	85
Benign uterine diseases group	100	94*	6*	93*	7*
Healthy control group	100	92*#	8*#	97*#	3*#

***Note:***

* indicated P<0.05, compared to the endometrial cancer group;

# indicated P>0.05 compared to the benign uterine diseases group.

### Methodological evaluation on the application of HE4, CA125 and HE4 in combination with CA125 in diagnosing endometrial cancer

The results demonstrated that, the sensitivity, Youden index and coincidence rate of diagnosing endometrial cancer with HE4 were superior to those with CA125. The sensitivity, Youden index, coincidence rate and negative predicted value of diagnosing endometrial cancer with HE4 in combination with CA125 were higher (Table-III).

**Table-III:** Comparison of performance of serum HE4 and CA125 in the diagnosis of endometrial cancer.

*Item*	*Sensitivity (%)*	*Specificity (%)*	*Youden index*	*Coincidence rate (%)*	*NPV (%)*	*PPV (%)*
HE4	57.2	95.8	0.518	79.8	76.5	89.6
CA125	51.7	93.9	0.457	77.1	73.7	85.2
HE4+CA125	73.2	91.2	0.641	83.5	83.4	84.8

NPV: Negative predictive value; PPV: Positive predictive value.

## DISCUSSION

Endometrial cancer refers to epitheliogenic malignant tumor initially occurring in endometrium, and adenocarcinoma derived from endometrial glands is the most common. Currently the pathogenesis and pathogenesis of endometrial cancer have not been clearly known. Most experts thought that endometrial cancer generates under the combined effect of inheritance, internal secretion and external environment.^11^ Though endometrial cancer manifests as increased menstrual volume, menostaxis and irregular vaginal bleeding, about 30% of patients who show no clinical symptoms have been at the advanced state when being diagnosed. Previously diagnostic curettage was regarded as the most common method for diagnosing endometrial cancer; however, it is a kind of invasive examination which is easy to cause physical and mental damages and moreover it is of small significance to clinical staging and has no values for treatment guidance and prediction of prognosis. Serum tumor markers which has advantages of small trauma and simple operation have gradually been the research hotspot.

CA125 is the major marker in the diagnosis and monitoring of endometrial cancer. But compared to ovarian cancer, the sensitivity and specificity of CA125 in diagnosing endometrial cancer are lower; hence it is only suitable for patients with advanced endometrial cancer or recurrent endometrial cancer. Therefore, finding out other tumor markets is of great significance. HE4 is considered as a novel marker for gynecolgical tumor. In malignant tumors especially malignant ovarian epithelial tumors, the expression level of HE4 increases, inducing the changes of serum HE4 level. Moore et al.^12^ found that the serum HE4 level of patients with endometrial cancer was significantly higher than that of healthy people and that the strong positive expression of HE4 in endometrial cancer tissues accounted for more than 90%, suggesting HE4 might be assistant to the diagnosis of endometrial cancer. Zhang Zhi et al.^13^ found that the level of HE4 in patients with endometrial cancer was obviously higher than that of patients with benign uterine diseases. The results of this study also suggested that the level of HE4 in the endometrial cancer group was much higher than that in the other two groups. HE4 is superior to other tumor markers in the diagnosis of early endometrial cancer. HE4 is more valuable than CA125 in the identification of benign endometrial cancer.^14^,^15^ The results of this study suggested that the area under ROC of diagnosis based on single detection of HE4 was larger than that based on single detection of CA125, which were similar to the studies mentioned above. Therefore, patients with higher HE4 level should be paid more attention. If diagnostic curettage does not indicate endometrial lesions, hysteroscopy can be carried out to reduce miss diagnosis and avoid delayed treatment.

The methodology evaluation on HE4 and CA125 demonstrated that, the sensitivity, Youden index and coincidence rate of diagnosis based on HE4 were superior to those of CA125. Some scholars proposed that, the sensitivity of CA125 in the diagnosis of endometrial cancer is unable to satisfy the diagnostic requirement in clinics, which requires further study. Angioli found that, the sensitivity of HE4 in the diagnosis of endometrial cancer was 59.4%, but that of CA125 was only 19.8%.^16^ Omer improved the sensitivity of diagnosing endometrial cancer to 84% by using detection of multiple serum markers such as HE4, CA125, CA15-3, carcino embryonie antigen and CA19-9,^17^ suggesting combined detection of HE4 and CA125 were expected to provide more information for the diagnosis of endometrial cancer to make up the deficiency of single detection. The research results suggested that, the sensitivity of diagnosing endometrial cancer with HE4 was much higher than that with CA125 was (51.7% vs 57.2%); the sensitivity of diagnosing endometrial cancer based on HE4 in combination with CA125 was 73.2%, much superior to the separate detection, which was consistent with previous research results. Therefore, it was considered that, CA125 in combination with HE4 had an important clinical value in the early diagnosis of endometrial cancer.

In addition, the analysis of ROC curve suggested that, the ROC-AUC value of the serum HE4 was higher than that of the CA125 and the ROC-AUC value of the combined detection was much higher than that of the detection of single index, which also suggested the superiority of the combined detection.

## CONCLUSION

The detection of serum markers is an important approach for the diagnosis of endometrial cancer. Serum HE4 level of patients with endometrial cancer is higher than that of healthy controls and patients with uterine benign lesions. The detection HE4 in combination with CA125 can more effectively identify benign and malignant endometrial cancer; hence it is worth promotion in clinical practice.

### Authors’ Contribution

**CHD:** Study design, data collection and analysis.

**PL & CL:** Manuscript preparation, drafting and revising.

**CHD & CL:** Review and final approval of manuscript.

## References

[1] Zhang H, Zhang SW (2012). The level of serum HE4 level of patients with ovarian cancer and its clinical value. Shaanxi Med J.

[2] Bignotti E, Ragnoli M, Zanotti L, Calza S, Falchetti M, Lonardi S (2011). Diagnostic and prognostic impact of serum HE4 detection in endometrial carcinoma patients. Br J Cancer.

[3] Zhang HR, Shang W, Ji LJ, Wang AM (2010). The value of serum HE4 detection in the diagnosis of ovarian malignant carcinoma. J Hebei Med Univ.

[4] Li J, Dowdy S, Tipton T, Podratz K, Lu WG, Xie X (2009). HE4 as a biomarker for ovarian and endometrial cancer management. Expert Rev Mol Diagn.

[5] Moore RG, Brown AK, Miller MC, Badgwell D, Lu Z, Allard WJ (2008). Utility of a novel Serum tumor biomarker HE4 in patients with endometrioid adenocarcinoma of the uterus. Gynecol Oncol.

[6] Kalogera E, Scholler N, Powless C, Weaver A, Drapkin R, Li J (2012). Correlation of serum HE4 with tumor size and myometrial invasion in endometrial cancer. Gynecol Oncol.

[7] Zhang AM, Zhang P (2012). The clinical value of the detection of HE4 and CA125 in the diagnosis of endometrial cancer. Chin J Obstet and Gynecol.

[8] Pearce CL, Templeman C, Rossing MA, Lee A, Near AM, Webb PM (2012). Association between endometriosis and risk of histological subtypes of ovarian cancer: a pooled analysis of case-control studies. Lancet Oncol.

[9] Jiang LN, Zhang HJ, Zhang HJ (2014). The study of HE4, CA125 and CA72-4 for differential diagnosis between ovarian endometriosis and ovarian cancer. Chin J Lab Diagn.

[10] Sandri MT, Bottari F, Franchi D, Boveri S, Candiani M, Ronzoni S (2013). Comparison of HE4, CA125 and ROMA algorithm in women with a pelvic mass: Correlation with pathological outcome. Gynecol Oncol.

[11] Bignotti E, Ragnoli M, Zanotti L, Calza S, Falchetti M, Lonardi S (2011). Diagnostic and prognostic impact of serum HE4 detection in endometrial carcinoma patients. Br J Cancer.

[12] Moore RG, Brown AK, Miller MC, Badgwell D, Lu Z, Allard WJ (2008). Utility of a novel serum tumor biomarker HE4 in patients with endometrioid adenocarcinoma of the uterus. Gynecol Oncol.

[13] Zhang Z, Chen HX, Xu XX (2016). The values of the combined detection of serum human epididymis protein E4, CA125 and CA199 in diagnosis of endometrial cancer. J Pract Med.

[14] Galgano MT, Hampton GM, Frierson HF (2006). Comprehensive analysis of HE4 expression in normal and malignant human tissues. Mod Pathol.

[15] Li J, Chen H, Mariani A, Chen D, Klatt E, Podratz K (2013). HE4 (WFDC2) promotes tumor growth in endometrial cancer cell lines. Int J Mol Sci.

[16] Angioli R, Plotti F, Capriglione S, Montera R, Danmiani P, Ricciardi R (2013). The role of novel biomarker HE4 in endometrial cancer: a case control prospective study. Tumour Biol.

[17] Omer B, Genc S, Takmaz O, Dirican A, Kusku-Kiraz Z, Berkman S (2013). The diagnostic role of human epididymis protein 4 and serum amyloid-A in endometrial cancer patients. Tumour Biol.

